# Group singing improves depression and life quality in patients with stable COPD: a randomized community-based trial in China

**DOI:** 10.1007/s11136-018-2063-5

**Published:** 2019-01-05

**Authors:** Hua Liu, Mei Song, Zhong-Hui Zhai, Rui-Jie Shi, Xiao-Lan Zhou

**Affiliations:** 1 College of Nursing, Xi’an Medical University, Xi’an, China; 2 Library of Xi’an Jiaotong University, Xi’an, China; 3 Nursing Department, The Fourth Military Medical University, Xi’an, China

**Keywords:** Group singing therapy, Quality of life, Depression, Chronic obstructive pulmonary disease

## Abstract

**Purpose:**

To explore the effects of group singing therapy on depression symptoms and quality of life of patients with stable chronic obstructive pulmonary disease (COPD).

**Methods:**

Patients with COPD were randomly allocated to intervention (*n* = 30) and control groups (*n* = 30). The intervention group received group singing therapy once a week for 24 sessions along with routine health education, whereas the control group only received the routine health education. All patients were administered the Hospital Anxiety and Depression Scale depression subscale (HADS-D) and the Clinical COPD Questionnaire (CCQ). Data were collected at baseline and at 1, 3, and 6 months.

**Results:**

Fifty-six participants completed this trial. Significant between-group differences were observed with respect to the main effect of group and time as well as the effect of group × time interaction on HADS-D score. The HADS-D score was significantly improved 1, 3, 6 months after group singing therapy. The CCQ total scores were significantly different between the two groups with respect to the main effect of group and time and the group × time interaction effect. Significantly better CCQ was detected in the intervention group at 3 months and 6 months after intervention.

**Conclusions:**

Group singing therapy reduces depressive symptoms and improves the quality of life of patients with stable COPD.

## Introduction

Chronic obstructive pulmonary disease (COPD) is characterized by progressive and irreversible airflow obstruction, and is associated with high morbidity and mortality [1]. Globally, COPD is the fourth leading cause of death and is projected to be the seventh highest contributor to the total disability-adjusted life years (DALYs) lost by the year 2030 [2, 3]; in addition, it ranks as the fourth leading cause of disability-adjusted life years in China [4]. Currently, COPD is a major public health problem owing to its detrimental effect on the health status of patients.

COPD is a chronic respiratory disease which is associated with multiple comorbidities including cardiovascular disease, skeletal muscle dysfunction, metabolic syndrome, osteoporosis, depression, and lung cancer [1]. However, depression is one of the most common comorbid conditions in patients with COPD. Moreover, COPD patients are more commonly affected by depression as compared to patients with other chronic conditions [2]. The prevalence of depression in people with COPD was shown to be twofold higher than that in people without COPD [5]. Owing to the considerable overlap between the somatic symptoms of depression and symptoms of COPD, the presence of this mental disease is liable to be unrecognized by COPD patients and more likely to be missed by their physicians as compared to patients with other comorbidities or depression alone [6]. Moreover, the stigma of mental ill-health can prevent people who may be suffering from depression from seeking care from a psychologist. Owing to these factors, depression may remain undetected, undertreated, or even untreated in patients with COPD. It can in turn lead to deterioration of COPD, since it may reduce patient compliance with COPD treatment. A meta-analysis of studies revealed that COPD patients with depression exhibit lower treatment adherence [7] and that depression can worsen the symptoms of COPD and make it harder to improve self-efficacy; in addition, depression was shown to increase the incidence of acute exacerbation and hospitalization [8, 9], impair the patient’s quality of life, worsen the prognosis [2], and lead to higher mortality [10]. Therefore, intervention for depression may play an important role in the management of patients with COPD. The combination of COPD and depression is projected to cause significant health problems in the next decade [5].

Studies have shown that identification and treatment of depressive symptoms in patients with COPD may have a favorable effect on mood, exercise tolerance, quality of life, and related symptoms [11–15]. Consequently, there is a need to identify suitable interventions to help COPD patients to manage depressive symptoms and to reduce the burden of COPD. The National Institute for Health and Care Excellence (NICE) recommends psychological and pharmacological therapies for treatment of depression based on solid systematic reviews or valuable experiences [16]. However, robust evidence of the efficacy of antidepressants in ameliorating depression or in improving the symptoms associated with COPD is largely lacking [17]. Moreover, some COPD patients may refuse pharmacological treatment owing to concerns pertaining to the side effect of antidepressant drugs. Recent studies have shown that pulmonary rehabilitation program can reduce the severity of depressive symptoms in COPD patients regardless of the disease stage, patients’ gender, age, or education level [11, 12]. Psychological interventions, such as cognitive behavioral therapy (CBT), have been widely used in depressed COPD patients; individualized or group CBT was shown to ameliorate depressive symptoms and prevent aggravation of COPD [11, 12]. Music therapy has been subjected to substantial research especially for treatment of chronic conditions, and it is one of the complementary or alternative therapies that belong to the category of “mind-body medicine”[18]. Several studies have documented its beneficial effects in patients with chronic diseases [19, 20]. Music therapy was shown to induce clinically significant changes in mood and symptoms of COPD patients [21]. Singing is an active music therapy and is widely used for treatment of physiological and psychological disorders [20, 22–25]. The IMPRESS British Thoracic Society Guidelines for Pulmonary Rehabilitation consider singing therapy as an adjuvant therapy [26]. Moreover, singing therapy may potentially improve physical health-related life quality and alleviate anxiety without any obvious side effects; however, conclusive evidence of the effect of singing therapy on clinical symptoms and the quality of life of COPD patients is yet to be obtained [27]. In a pilot study, singing therapy was shown to enhance lung function, reduce anxiety, and boost self-esteem of patients with COPD; however, the improvement in quality of life was not statistically significant [28]. Two previous studies suggest that singing in group improves the quality of life, ameliorates anxiety levels, and induces a feeling of well-being and social support in patients with chronic respiratory diseases [24, 25]. A cohort of COPD patients who were recruited to a new community-based singing group, which met weekly for over 1 year, revealed significant reduction in Hospital Anxiety and Depression Scale (HADS) anxiety score but not in HADS depression score (HADS-D) after 1 year [29].

Reports have shown that music facilitates expression of emotions among participants [30], which may help uplift the mood. In addition, singing is a particular type of respiratory exercise that demands repetitive diaphragm contractions, followed by sustained contractions of expiratory muscles against semi-closed vocal cords during expirations. This training involving breathing control and respiratory muscle exertion may potentially improve the pulmonary function and help alleviate symptoms of COPD [31]. It is noteworthy that depression showed a strong correlation with COPD symptoms and quality of life. COPD patients with depression experience more frequent exacerbations, and have higher health care resource utilization and reduced health-related quality of life [8, 9]. Depression is an independent risk factor for COPD [1], and depression influences treatment compliance and worsens the condition [7]. Therefore, it is particularly important to improve depressive symptoms in COPD patients. However, no studies have investigated the effect of music therapy on depressive symptoms and the quality of life of patients with stable COPD and comorbid depression. We hypothesized that group singing practice could decrease depressive symptoms and improve the quality of life of these patients.

## Methods

### Study design and participants

**Fig. 1:**
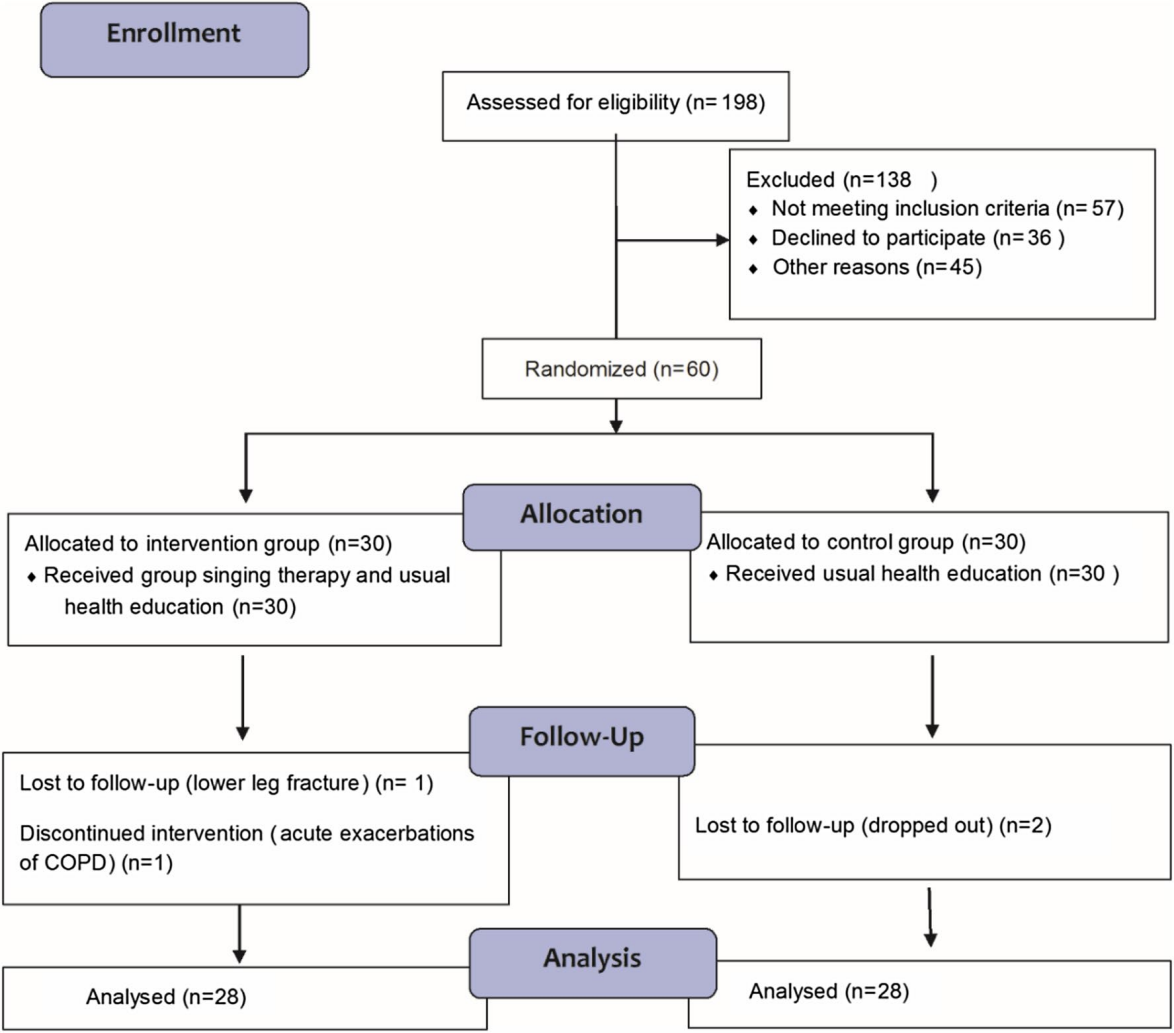
Flow diagram of the study

This was a prospective, randomized controlled trial. Participants were recruited from four large communities in Xi’an, Shaanxi Province of China from April to October, 2016. A total of 198 patients were screened; of these, 60 were found to be eligible and consented to participate in the study. The participants were counseled about the purpose of the study, i.e., to exercise the respiratory muscles and prevent recurrent attacks. Patients were randomly allocated to the intervention or control group using a random number table. Single blind method was used in the study. The control group received routine health education, while the intervention group received group singing therapy in addition to routine health education. During the study, two participants in the intervention group withdrew because of deterioration in health status and two participants in the control group were lost to follow-up. A total of 56 patients were eventually included in the analysis. With the consent of the participants, paper questionnaires were distributed and completed by the participants within 20 min at their respective homes. Data were collected and analyzed at baseline and at 1, 3, and 6 months thereafter [32, 33]. The time-points of 1 month and 3 months represent the early stage and middle stage of intervention, respectively. The flow diagram of the study is depicted in Fig. 1.

Inclusion criteria were as follows: (1) patients with COPD, diagnosed according to the criteria of Global initiative for Chronic Obstructive Lung Disease (Global Strategy for the Diagnosis, Management and prevention of Chronic Obstructive Pulmonary Disease) [34]; (2) patients with clinically stable disease and no exacerbations in the last 30 days; (3) Hospital Anxiety and Depression Scale evaluation-depression subscale score ≥ 8. The exclusion criteria were (1) inability to walk independently (*n* = 9); (2) severe cognitive (*n* = 2) or hearing impairment (*n* = 3), cerebrovascular disease (*n* = 6), and cardiopulmonary dysfunction (*n* = 15); (3) patients on antidepressants (*n* = 4) and other mood-focused therapies (*n* = 6); (4) patient refusal to participate in the study (*n* = 36).

This study was approved by the Medical Ethics Committee of the Xi’an Medical University (No. XMU16-046). Written informed consent was obtained from all participants prior to their enrolment. Participants were free to opt out of the study at any time.

### Interventions

#### Control group

Patients in the control group were provided with routine health education, which included eight sessions of COPD education (30 min each). Community nurses explained the COPD disease-specific information and preventive information. Smoking cessation is the most important measure to prevent progression of disease and elimination of this risk factor is an important step for prevention and control of COPD. Other education topics included change in unhealthy daily lifestyle choices or habits, significance of respiratory function exercises, and demonstration of how to perform these exercises. Patients were instructed to perform respiratory exercises for 10 min at a time, twice a day, and were asked to participate in physical exercises (such as walking, jogging) as much as possible according to their physical condition. In addition, a health education handbook was offered to all participants. To balance the amount of attention received by both groups, patients in control group were asked to gather once a week for experience sharing after completion of eight sessions of health education.

#### Intervention group

In addition to routine health education, the intervention group received group singing therapy once a week for 24 sessions (each session lasted approximately 60 min). The participants gathered in a large room at a community recreation center and the activity was coordinated by a music therapist and a community nurse. Participants were free to sit, stand, or lean against the wall according to their physical condition. The intervention protocol was implemented as follows:

- *Relaxation exercises* The community nurse guided the patients to perform relaxation exercises for the neck and upper abdominal muscles. Neck muscle relaxation required the patient to lower the head and try to position the chin against the chest for l0 s. Upper abdominal muscle relaxation required the patient to gradually lift up the upper limbs and inhale deeply, and then put down slowly the upper limbs and exhale slowly while breathing evenly; the relaxation exercises were repeated several times (5 min).
- *Respiratory exercises* The participants were instructed to practice two specific breathing techniques, i.e., pursed-lip breathing and diaphragmatic breathing. Participants were initially encouraged to perform deep inhalation slowly through the nose followed by slow exhalation through pursed lips while paying attention to the upper abdominal muscle movements at the same time (10 min).
- *Vocalization exercises* The purpose of this exercise was to train the patients with resonance organ function, and to achieve breath and voice coordination. The patients were asked to vocalize with slowly whispered hum and loud hum, respectively (e.g., repeated practice of mi, mi, ma, ma scales) (15 min).
- *Singing exercises* This was the most important part of group singing therapy. The participants sang one song selected from a list of participant-chosen music selection on each occasion (folk, classical, familiar, and happy songs). At first the music therapist taught everybody to sing the song several times; subsequently, the participants sang the song accompanied by music therapist on the piano. Finally, the patients copied the song in MP5 format and practiced singing at home for 30 min every day (30 min).

### Instruments

Data were collected by means of self-administered questionnaires. The HADS-D subscale was used for assessment of depressive symptoms. This scale was developed by Zigmond et al. [35] and includes seven items. Each item is scored on a four-point (0–3) scale; the total score ranges from 0 to 21. A score of 8 or higher is indicative of depression. HADS has been shown to have a high sensitivity and specificity in Chinese patients (Cronbach’s alpha for HADS-D: 0.79) [36].

The Clinical COPD Questionnaire (CCQ) was used to assess the disease-specific quality of life [37]. The questionnaire includes symptoms (four items), mental state (two items), and functional status (four items) and is graded on a 7-point scale from 0 (very good control) to 6 (extremely poor control). Higher scores indicate worse health status or poorer control of COPD. The instrument was shown to have a high level of internal consistency (Cronbach’s alpha 0.91). Internal consistency for the symptoms, mental state, and functional state domain were 0.78, 0.80, and 0.89, respectively.

### Data analysis

Data were processed using Epidata (version 3.1; Lauritsen & Bruus, Odense, Denmark) software. All statistical analyses were performed using SPSS (version 13.0; SPSS Inc., Chicago, IL, USA). Descriptive statistics were generated for demographic variables. Between-group differences with respect to baseline characteristics were assessed using the Chi-squared test and *t* test. Repeated measures ANOVA and *t* test were used to compare variables among groups. *P* values < 0.05 were considered indicative of statistically significant between-group difference.

## Results

### Baseline demographic and clinical characteristics data

Data pertaining to demographic variables (gender, age, educational level), history of smoking, duration of disease, and COPD stage were obtained from a structured questionnaire and pulmonary function tests. A total of 60 subjects participated in the current study, of whom 56 (50 males and 6 females) completed the study and were included in the analysis. Figure 1 displays the CONSORT flow chart. Table 1 summarizes the baseline demographic and clinical characteristics. The variables included gender, age, education level, smoking history, COPD stage, and duration of disease. The mean age of participants was 63.51 ± 4.72 years. More than 57% participants received college/university education. More than 87% participants had a history of smoking. More than half of the patients had moderate COPD and the distribution of patients with disease duration of < 5, 5–10, and ≥ 10 years was comparable in the two groups. No significant between-group differences were observed with respect to demographic and clinical characteristics.

**Table 1:** Baseline demographic and clinical characteristics of subjects

Variable	Intervention group	Control group	*p* values
Gender			
Male [*n* (%)]	24 (85.71%)	26 (92.86%)	0.669^a^
Female [*n* (%)]	4 (14.29%)	2 (7.14)	
Age (years)	63.85 ± 4.25	63.30 ± 5.49	0.742^b^
Education level			
Elementary school or under [*n* (%)]	5 (17.86%)	6 (21.43%)	0.864^c^
Secondary school [*n* (%)]	6 (21.43%)	7 (25.00%)	
College/university [*n* (%)]	17 (60.71%)	15 (53.57%)	
Smoking history			
Yes [*n* (%)]	25 (89.29%)	24 (85.71%)	1.0^a^
No [*n* (%)]	3 (10.71%)	4 (14.29%)	
COPD stage			
Mild [*n* (%)]	6 (21.43%)	8 (28.57%)	0.814^c^
Moderate [*n* (%)]	17 (60.71%)	15 (53.57%)	
Severe [*n* (%)]	5 (17.86%)	5 (17.86%)	
Duration of disease (years)			
≤ 5 [*n* (%)]	8 (28.57%)	9 (32.14%)	0.889^c^
5–10 [*n* (%)]	11 (39.29%)	12 (42.86%)	
≥ 10 [*n* (%)]	9 (32.14%)	7 (25.00%)	

^a^Fisher’s exact probability test

^b^Independent sample *t* test

^c^Pearson’s Chi-squared test

### Comparison of two groups in terms of depression

**Fig. 2:**
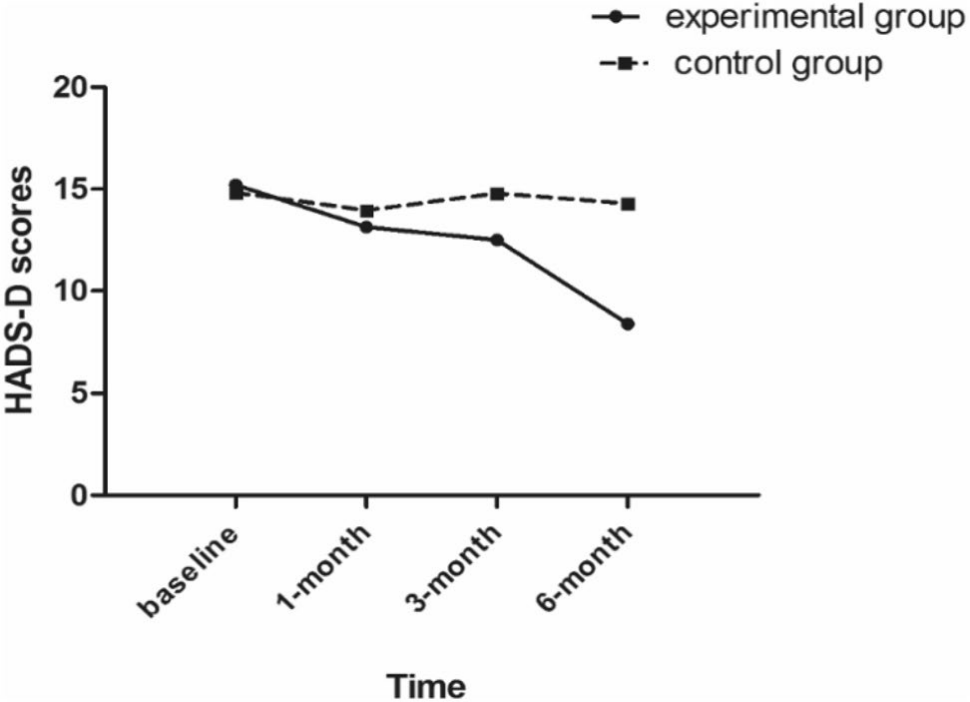
The trend of scores in the Hospital Anxiety and Depression Scale depression subscale questionnaire at different time-points in the experimental and control groups

The depression scores showed significant between-group differences along with the time, which was represented by a group × time interaction effect (*F*_(1,54)_ = 38.19, *p* < 0.001, Partial *η*^2^ = 0.414; *F*_(3, 162)_ = 96.95, *p* < 0.001, Partial *η*^2^ = 0.642; *F*_(3, 162)_ = 77.67, *p* < 0.001, Partial *η*^2^ = 0.590) (Table 2), and the depression scores in the experimental group showed a significant declining trend along with the time. These findings indicate that the beneficial effects of group singing therapy increased with increase in the duration of therapy (Fig. 2). The results of HADS-D score in the two groups at different time-points are shown in Table 3. No significant between-group difference was observed with respect to baseline measures (*t*_55_ = 0.790, *p* > 0.05). The HADS-D scores in the control group were relatively stable over the duration of the study. In contrast, the intervention group exhibited a significant improvement in HADS-D scores at 1 month (*t*_55_ = − 2.211, *p* < 0.05), 3 months (*t*_55_ = − 6.358, *p* < 0.001), and 6 months (t55 = − 11.621, *p* < 0.001) after intervention.

**Table 2:** Repeated measures ANOVA of the depression score

Source	Type III sum of squares	df	Mean square	*F* values	*p* values	Partial *η*^2^
Group	259.29	1	259.29	38.19	< 0.001	0.414
Time	388.98	3	129.66	96.95	< 0.001	0.642
Group × time	311.62	3	103.87	77.67	< 0.001	0.590
Within-group error	216.65	162	1.34			
Between-group error	366.67	54	6.79			

**Table 3:** Comparison of depression scores between the two groups at various time-points

	Experiment group (*n* = 28)		Control group (*n* = 28)		*t*	*p* values
	M	SD	M	SD		
Baseline	15.21	2.02	14.82	1.68	0.790	0.218
1-month	13.14	1.53	13.96	1.23	− 2.211	0.031
3-month	12.50	1.17	14.79	1.50	− 6.358	< 0.001
6-month	8.39	1.75	14.29	2.03	− 11.621	< 0.001

*M* mean, *SD* standard deviation

### Comparison of two groups in terms of CCQ

**Fig. 3:**
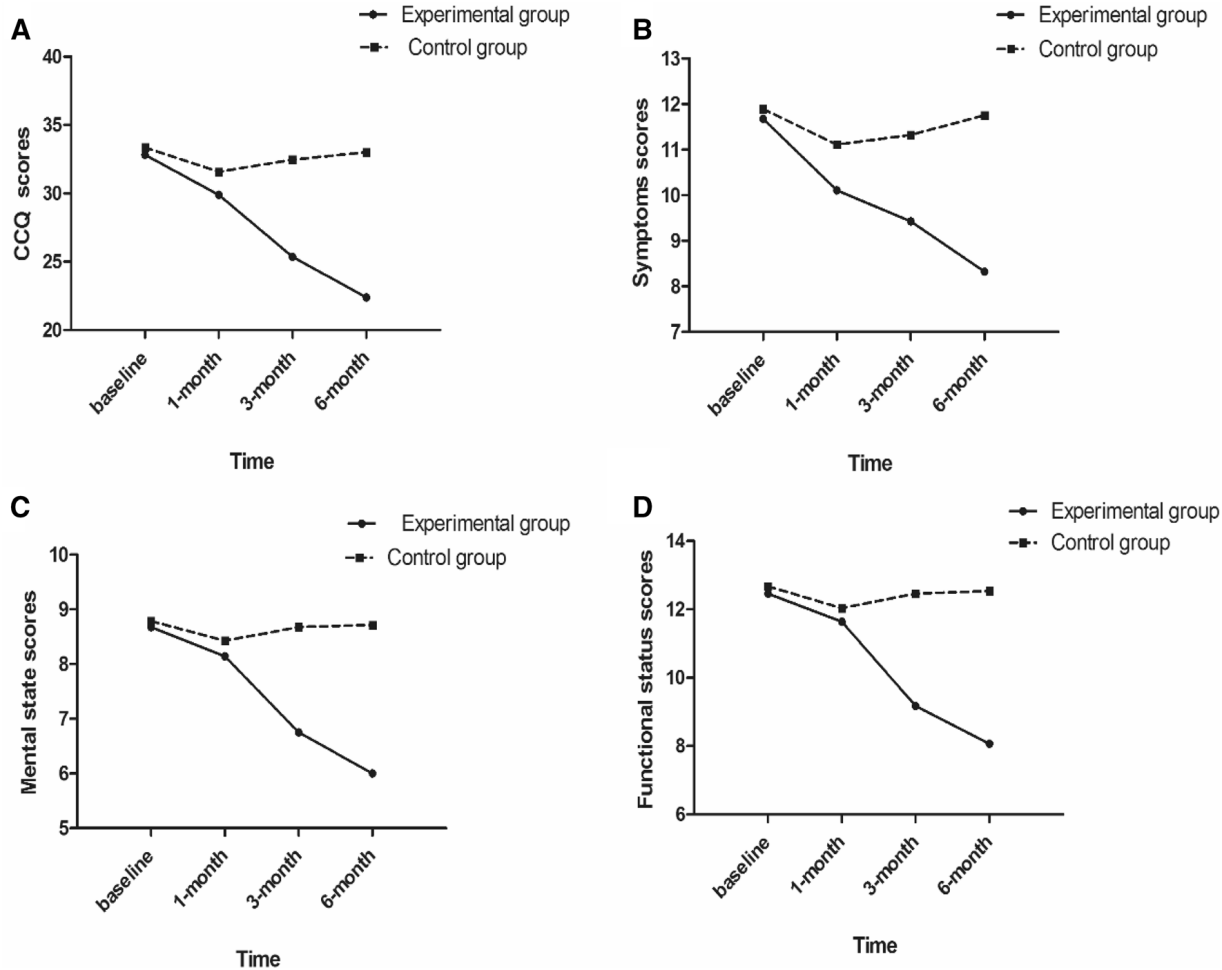
The trend of scores in the Clinical COPD Questionnaire (CCQ) at various time-points in the experimental and control groups. **a** CCQ total score; **b** symptoms score; **c** mental state score; **d** functional status score

Significant differences were also observed with respect to the main effect of group and time as well as the effect of group × time interaction on CCQ scores [the total score (*F*_(1,54)_ = 47.00, *p* < 0.001, Partial *η*^2^ = 0.465; *F*_(3,162)_ = 42.02, *p* < 0.001, Partial *η*^2^ = 0.438; *F*_(3,162)_ = 42.91, *p* < 0.001, Partial *η*^2^ = 0.440) and symptoms score (*F*_(1,54)_ = 27.44, *p* < 0.001, Partial *η*^2^ = 0.337; *F*_(3,162)_ = 13.37, *p* < 0.001, Partial *η*^2^ = 0.198; *F*_(3,162)_ = 11.00, *p* < 0.001, Partial *η*^2^ = 0.169) and mental state score (*F*_(1,54)_ = 18.99, *p* < 0.001, Partial *η*^2^ = 0.260; *F*_(3,162)_ = 14.49, *p* < 0.001, Partial *η*^2^ = 0.212; *F*_(3,162)_ = 15.79, *p* < 0.001, Partial *η*^2^ = 0.226) and functional status score (*F*_(1,54)_ = 37.20, *p* < 0.001, Partial *η*^2^ = 0.408; *F*_(3,162)_ = 33.17, *p* < 0.001, Partial *η*^2^ = 0.381; *F*_(3,162)_ = 38.66, *p* < 0.001, Partial *η*^2^ = 0.417)] (Table 4), The CCQ scores in the experimental group showed a significant declining trend at 3-month and 6-month time-points (Fig. 3). The CCQ scores for the experimental and control groups are shown in Table 5. No significant between-group difference was observed at baseline and at the 1-month time-point and no significant change in health-related quality of life (HRQL) over time was observed in the control group. However, a significant difference was detected at 3-month and 6-month time-points in the experimental group. The total scores in the experimental group were significantly lower than those in the control group at different time-points; these findings indicate a beneficial effect of group singing therapy on HRQL of patients with COPD.

**Table 4:** Repeated measures ANOVA of the CCQ scores

Source	Type III sum of squares	Df	Mean square	F values	*p* values	Partial *η*^2^
CCQ total score						
Group	1390.02	1	1390.02	47.00	< 0.001	0.465
Time	925.50	3	308.50	42.02	< 0.001	0.438
Group × time	935.77	3	311.92	42.91	< 0.001	0.440
Within-group error	1189.23	162	7.34			
Between-group error	1596.91	54	29.57			
Symptoms score						
Group	149.50	1	149.50	27.44	< 0.001	0.337
Time	97.12	3	32.37	13.37	< 0.001	0.198
Group × time	79.87	3	26.62	11.00	< 0.001	0.169
Within-group error	392.26	162	2.42			
Between-group error	294.21	54	5.45			
Mental state score						
Group	88.75	1	88.75	18.99	< 0.001	0.260
Time	62.19	3	20.73	14.49	< 0.001	0.212
Group × time	67.76	3	22.59	15.79	< 0.001	0.226
Within-group error	231.80	162	1.43			
Between-group error	252.38	54	4.67			
Functional status score						
Group	238.22	1	238.22	37.20	< 0.001	0.408
Time	166.66	3	55.55	33.17	< 0.001	0.381
Group × Time	194.26	3	64.75	38.66	< 0.001	0.417
Within-group error	271.33	162	1.68			
Between-group error	345.78	54	6.40			

**Table 5:** Comparison of CCQ scores in the two groups at various time-points

	Experiment group (*n* = 28)		Control group (*n* = 28)		*t*	*p* values
	M	SD	M	SD		
CCQ total score						
Baseline	32.82	3.54	33.36	2.98	– 0.612	0.543
1-month	29.89	4.18	31.57	3.93	– 1.547	0.128
3-month	25.36	4.31	32.46	3.26	– 6.964	< 0.001
6-month	22.39	3.52	33.00	2.69	– 12.660	< 0.001
Symptoms score						
Baseline	11.68	1.66	11.89	1.52	– 0.504	0.616
1-month	10.11	2.15	11.11	1.62	– 1.967	0.055
3-month	9.43	1.95	11.32	1.72	– 3.848	< 0.001
6-month	8.32	1.87	11.75	1.69	– 7.202	< 0.001
Mental state score						
Baseline	8.68	1.70	8.79	1.62	– 0.241	0.810
1-month	8.14	1.60	8.43	1.48	– 0.694	0.491
3-month	6.75	1.48	8.68	1.63	– 4.627	< 0.001
6-month	6.00	0.98	8.71	1.36	– 8.577	< 0.001
Functional status score						
Baseline	12.46	2.06	12.57	1.66	– 0.214	0.831
1-month	11.64	1.25	12.03	1.75	– 0.965	0.339
3-month	9.18	1.91	12.46	1.55	– 7.075	< 0.001
6-month	8.07	1.63	12.54	1.57	– 10.418	< 0.001

*M* mean, *SD* standard deviation

## Discussion

Our results indicate a beneficial effect of group singing therapy on mild to severe COPD following depression in stable clinical conditions, not only with respect to alleviation of depressive symptoms, but also with respect to improvement in the quality of life. However, no significant effect was observed in the control group.

The results of this study suggest that group singing therapy may alleviate depression in patients with COPD. Our findings are not consistent with those of other studies [24, 25], which showed that singing therapy has no obvious effect in improving depression but that it relieves anxiety. The difference may be attributable to different song types. In this study, we chose the folk, classical, familiar, and happy songs, which are easy to learn and may have an uplifting effect. Studies have shown that singing of songs with cheerful rhythm makes people feel excited and modulates brain cortex, limbic system, brainstem reticular formation, endocrine system, and nervous system [38]. It was also shown that singing can be a joyful and uplifting experience for participants owing to generation of a sense of positive mood, happiness, and enjoyment [23, 39]. However, no change in depressive symptoms was observed in the control group, which suggests that routine health education had limited impact on the psychological health of COPD patients. The presence of the symptoms of dyspnea or depression in COPD patients is more likely to result in social isolation and loneliness [9]. Although the equal amount of attention was given to the control group by experience sharing, the communication opportunity provided by the group singing therapy may be more positive and the subjects were likely to express their active emotions. For these reasons, group singing therapy could ease the depression of COPD patients.

As there is no cure for COPD, a major goal of treatment is to improve health-related quality of life in these patients. Mood disorders, lower levels of exercise tolerance, and dyspnea were shown to affect the quality of life and health status of COPD patients [12]. In addition, a recent study showed a significant negative association between dyspnea and health status of COPD patients [40]. In the experiment group, no significant differences were observed at the 1-month time-point, which may be attributable to the short duration of intervention. However, a significant downward trend was observed at the 3-month and 6-month time-points; the participants benefited from the intervention as it provided an environment that was conducive to sharing of interests and experiences. Moreover, the breathing exercises, vocal exercises, and singing exercises also expand the lung capacity, increase alveolar ventilation, reduce residual volume, and increase the strength of lower chest muscles, diaphragm, and abdominal muscles [38]; moreover, this increases the ability for clearance of sputum and activity endurance, and relieve the symptoms of cough and dyspnea [25, 41]. In the control group, CCQ scores at the 1-month time-point showed a downward tendency; however, at the 3-month and 6-month time-points, the CCQ scores showed a significant upward trend. These results indicate that routine health education may improve quality of life [42], but this improvement is not maintained for a long time; a major reason might be associated with depression, which may lead to poor adherence to exercise [7]. COPD patients with depressive symptoms often develop a considerable degree of hopelessness and pessimism [6]. Consequently, this makes it difficult for patients to develop self-management ability and improve the quality of their life.

Our findings have shown that group singing therapy can reduce symptoms of depression and improve the quality of life of patients with stable COPD. This may be attributable to the several factors. All participants in this study were older than 55 years; most of them were retired and had much free time to actively participate in the study. Moreover, the participants had a history COPD since approximately 10 years; therefore, they were motivated to control the disease. Although this was a fairly representative sample of COPD patients, certain categories of patients may have been missed due to the relatively small sample size. Besides, due efforts were made during the questionnaire survey to minimize the possibility of response bias. For example, the word “depression” was not used in the HADS-D questionnaire to prevent the participants from guessing the purpose of the study.

## Strengths and limitations

The study provided a direct evidence of the beneficial effect of group singing therapy on COPD patients with depression for the first time. Considering the high prevalence and low detection rate of depression among COPD patients, our study provides a beneficial and convenient therapy for COPD patients both for alleviating depression and for improving the quality of life. There are several advantages of singing: (1) is a joyful and interesting task and should be easy for patients with COPD to continually engage in the therapy; (2) has no obvious side effects, is of low-cost intervention, and is easy to be carried out for free; (3) helps in exercising the respiratory function, improves vital capacity and cardiac-pulmonary function, and is well accepted by patients with COPD; and (4) inspires confidence and love for life.

However, there are several limitations of the study. The main limitation is that patients with very severe COPD were not included. It remains unknown whether patients with severe disease can tolerate group singing therapy and whether singing therapy interventions could offer benefit for these patients. Second, this study lasted for only 6 months and it is not known whether the results noted here could be maintained over time. Third, detailed data pertaining to treatment adherence were not collected as there are no appropriate rating scales.

## Relevance for clinical practice

Our findings illustrate that group singing therapy may help reduce symptoms of depression and improve the quality of life of patients with stable COPD. A collaborative clinical team, including multi-disciplinary healthcare professionals and community health service, should pay close attention to and manage symptoms of depression in people with COPD. In clinical practice, there is a need for routine assessment and early detection of symptoms of depression in patients with COPD. Group singing therapy is well accepted by the participants, and nurses may instruct patients with stable COPD to practice it at home or in the community. The enhanced efficacy of the intervention over time can potentially prevent acute exacerbations and improve health-related quality of life among community population. More effort is needed to focus on detailed clinical practice method.

## Conclusions

It can be concluded that the group singing therapy may help decrease depressive symptoms and improve quality of life. The beneficial effects of group singing therapy increased with increase in the duration of therapy. Furthermore, it is an enjoyable experience and well accepted by the participants, which may facilitate its use as a valuable complementary therapeutic method.
